# Reconceptualising depression along the endogeneus-reactive spectrum: are different genes involved in depression depending on presence vs absence of exposure to stress?

**DOI:** 10.1192/j.eurpsy.2024.99

**Published:** 2024-08-27

**Authors:** X. Gonda

**Affiliations:** Department of Psychiatry and Psychotherapy, Semmelweis University, Budapest, Hungary

## Abstract

**Abstract:**

Depression is a complex and highly heterogeneous disorder with an omnigenic and multifactorial background. This diversity is obvious not only in its symptomatic manifestation but also in its neurobiological underpinnings which is one potential factor contributing to the high observed rate of treatment resistance. Thus, subtyping depressions, understanding their distinct neurobiological and genetic background, and potentially developing biomarkers aiding their differential diagnosis may bring us one step closer to more effective treatment. The present talk will overview the different etiological factors contributing to the emergence of depression along an endogenous-reactive continuum, the contributory roles of different types of stress, different genes involved in distinct processes, and the potential consequences of conceptualising, diagnosing and treating depressions developing in the context or independently of current stress.

**Disclosure of Interest:**

None Declared

